# Targeting inhibitor of apoptosis proteins in combination with ErbB antagonists in breast cancer

**DOI:** 10.1186/bcr2328

**Published:** 2009-06-29

**Authors:** Fiona M Foster, Thomas W Owens, Jolanta Tanianis-Hughes, Robert B Clarke, Keith Brennan, Nigel J Bundred, Charles H Streuli

**Affiliations:** 1 Faculty of Life Sciences, University of Manchester, Oxford Road, Manchester M13 9PT, UK; 2 Faculty of Medical and Human Sciences, University of Manchester, Manchester M20 4BX, UK; 3 Department of Surgery, South Manchester University Hospitals Trust, Wythenshawe Hospital, Southmoor Road, Manchester M23 9LT, UK

## Abstract

**Introduction:**

Inhibitor of apoptosis (IAPs) proteins are a family of proteins that can block apoptosis in normal cells and have been suggested to cause resistance to apoptosis in cancer. Overexpression of oncogenic receptor tyrosine kinases is common in breast cancer; in particular 20% of all cases show elevated Her2. Despite clinical success with the use of targeted therapies, such as Trastuzumab, only up to 35% of Her2-positive patients initially respond. We reasoned that IAP-mediated apoptosis resistance might contribute to this insensitivity to receptor tyrosine kinase therapy, in particular ErbB antagonists. Here we examine the levels of IAPs in breast cancer and evaluate whether targeting IAPs can enhance apoptosis in response to growth factor receptor antagonists and TRAIL.

**Methods:**

IAP levels were examined in a breast cancer cell line panel and in patient samples. IAPs were inhibited using siRNA or cell permeable mimetics of endogenous inhibitors. Cells were then exposed to TRAIL, Trastuzumab, Lapatinib, or Gefitinib for 48 hours. Examining nuclear morphology and staining for cleaved caspase 3 was used to score apoptosis. Proliferation was examined by Ki67 staining.

**Results:**

Four members of the IAP family, Survivin, XIAP, cIAP1 and cIAP2, were all expressed to varying extents in breast cancer cell lines or tumours. MDAMB468, BT474 and BT20 cells all expressed XIAP to varying extents. Depleting the cells of XIAP overcame the intrinsic resistance of BT20 and MDAMB468 cells to TRAIL. Moreover, siRNA-based depletion of XIAP or use of a Smac mimetic to target multiple IAPs increased apoptosis in response to the ErbB antagonists, Trastuzumab, Lapatinib or Gefitinib in Her2-overexpressing BT474 cells, or Gefitinib in EGFR-overexpressing MDAMB468 cells.

**Conclusions:**

The novel findings of this study are that multiple IAPs are concomitantly expressed in breast cancers, and that, in combination with clinically relevant Her2 treatments, IAP antagonists promote apoptosis and reduce the cell turnover index of breast cancers. We also show that combination therapy of IAP antagonists with some pro-apoptotic agents (for example, TRAIL) enhances apoptosis of breast cancer cells. In some cases (for example, MDAMB468 cells), the enhanced apoptosis is profound.

## Introduction

One of the major hurdles in the treatment of breast cancer is resistance to therapy, resulting in tumour recurrence and patient mortality. A potential mechanism by which cancer cells escape drug-induced cell death is their intrinsic, or indeed acquired, resistance to apoptosis. Resistance may result from a dysregulation of anti-apoptotic inhibitor of apoptosis (IAPs) proteins or Bcl-2 proteins, which are therefore considered novel therapeutic targets for cancer [1-3]. There has been little work, however, to establish whether antagonists of endogenous anti-apoptotic proteins, such as IAPs, can improve the efficacy of targeted therapies for breast cancer. In the present article we conduct proof-of-principle studies to determine whether IAPs contribute to the apoptosis resistance of breast cancer cells to TNF-related apoptosis-inducing ligand (TRAIL) and ErbB antagonists.

Apoptosis mainly occurs through one of two pathways, the extrinsic pathway or the intrinsic pathway. The extrinsic pathway is activated by death ligands such as TRAIL, while the intrinsic pathway occurs in response to cell stresses such as growth factor withdrawal or DNA damage. Following activation of either apoptotic pathway, the caspase family of proteases execute cells through their proteolytic activity. IAPs can in turn negatively regulate caspases, blocking apoptosis.

XIAP (BIRC4) is the most potent caspase inhibitor in the IAP family: it binds to and inhibits active caspases 3, 7 and 9, and additionally ubiquitinates them [4-7]. Two further IAPs, cIAP1 (BIRC2) and cIAP2 (BIRC3), also bind caspases but do not directly inhibit them, instead inducing their proteasomal degradation [8,9].

The IAPs themselves are controlled at several levels, including the release of a pro-apoptotic factor – second mitochondrial activator of caspases (Smac) – from the mitochondria during apoptosis. Smac displaces caspases from XIAP, thereby preventing the inhibitory function of XIAP and promoting caspase activity [10]. The cIAPs achieve part of their anti-apoptotic function by binding to and ubiquitinating Smac, freeing XIAP to suppress caspase activity [8,9].

Since IAPs and their regulators act in a concerted manner during apoptosis, their dysregulation can increase the threshold for apoptosis in cancer, thereby contributing to disease progression [2]. For example, Survivin is normally only expressed during mitosis in adult cells, but is dramatically upregulated in many cancers leading to a poor prognosis for recurrence-free survival [11-13]. Overexpression of the other IAP family members in cancer also occurs but is not as clearcut as for Survivin. XIAP is ubiquitous in normal tissues, and is elevated in some cancers including renal, acute myeloid leukaemia and bladder cancer [14-16]. The correlation between elevated XIAP levels and clinical outcome, however, is not straightforward since its overexpression correlates with disease severity in acute myeloid leukaemia but not in lung cancer or prostate cancer [17,18]. There are less data on cIAPs, although chromosomal amplification of 11q21–q23, which encodes both cIAP1 and cIAP2, is observed in oesophageal squamous cell carcinomas and cIAP2-activating translocations can occur in some B-cell lymphomas [1,19]. cIAP1 also has oncogenic potential, as it has the ability to transform liver cells into hepatomas in combination with the oncogene *Yap *[20].

The anti-apoptotic function of IAPs suggests that they are attractive clinical targets (whether or not their levels are altered in cancer). Antisense oligodeoxynucleotides directed against XIAP and Survivin, and Smac mimetics targeting IAPs, are in phase I and phase I/II clinical trials [21-23].

In breast cancer, most of the work on IAPs has focused on Survivin. There are only a few reports examining the other IAPs, and this is in a few cell lines [24-26]. Studies have shown that inhibiting IAPs augments the apoptotic effect of chemotherapeutics (for example, cisplatin and etoposide) [27-29]. Few studies, however, have examined whether targeting IAPs can improve the efficacy of newer targeted therapies against oncogenic growth factor receptors in breast cancer [30,31].

In the present study we examine IAP expression in 14 commonly used breast cancer cell lines compared with a nonmalignant control line. We show that inhibiting IAPs either using siRNA directed against XIAP or using a Smac mimetic overcomes the intrinsic resistance of some of the breast cancer cell lines to both TRAIL and targeted therapies against ErbB receptors. Inhibiting IAPs may be clinically relevant since the IAP expression profile is altered in tumour biopsy samples.

## Materials and methods

### Antibodies

The following primary antibodies were used: cIAP2 and Desmoplakin (Abcam, Cambridge, UK); monoclonal XIAP (clone 2F1) and calnexin (Bioquote Ltd, York, UK); Ki67-FITC (BD Transduction Labs, Franklin Lakes, NJ, USA); phospho-Erk and oestrogen receptor alpha (Cell Signaling, Beverly, MA, USA); Cytokeratin 8/18 (Progen, Heidelberg, Germany); Survivin (Novus Biologicals, Littleton, CO, USA); cleaved caspase 3 (R&D Systems, Abingdon, UK); Erk, mouse monoclonal epidermal growth factor receptor (EGFR) (R1) and rabbit polyclonal EGFR (Santa Cruz, Santa Cruz, CA, USA); and Her2 (Upstate, Millipore, MA, USA). Anti-cIAP1 was a generous gift from J Silke [32].

### Cell culture

HS578T cells, MDAMB468 cells, CAL51B cells, MDAMB231 cells, SKBR3 cells, MCF7 cells, Zr-75-1 cells, BT474 cells, BT20 cells and T47D cells were all grown in DMEM supplemented with 10% FCS, 2 mM glutamine, penicillin-streptomycin. PMC42 cells were grown in RPMI with 10% FCS, 2 mM glutamine, penicillin-streptomycin. The receptor status of the cell lines was confirmed by western blotting (data not shown).

The MCF10 progression panel cells (from K Brennan and originally from the Karmanos Cancer Institute, Detroit, MI, USA) were grown in DMEM/F12 media supplemented with 5% horse serum, 2 mM glutamine, penicillin-streptomycin, with additional 5 μg/ml hydrocortisone, 10 μg/ml insulin, and 20 ng/ml epidermal growth factor for the MCF10a cells, MCF10neoT cells, and MCF10AT1 cells.

### Tumour samples

Approval to remove normal and tumorigenic human breast tissues during reduction mammoplasty and from pathologic samples, respectively, was obtained from the Manchester Local Research Ethics Committees. Written informed consent was obtained from the patients before surgery.

The receptor status of the tumour samples was determined clinically. Frozen sections were diced with a clean razor blade before lysis in RIPA buffer (150 mM NaCl, 50 mM Tris, pH 7.4, 5 mM ethylenediamine tetraacetic acid, 1% NP-40, 1% deoxycholate, 10 mM NaF, 1 mM Na_3_VO_4_, 1× Protease Inhibitor Cocktail (Calbiochem, San Diego, CA, USA)) and protein expression was analysed by immunoblotting.

### Cell lysis and immunoblotting

Cells were routinely lysed at 80% confluency in RIPA buffer. Proteins were separated by SDS-PAGE and transferred to nitrocellulose membranes, and were subsequently detected using the relevant primary antibody and appropriate secondary antibody (1:10,000). Secondary antibody detection was performed with either peroxidase-conjugated antibodies from Jackson Immunoresearch (West Grove, PA, USA) and Pierce SuperSignal West Pico Chemiluminescent Substrate (Rockford, IL, USA), or IR-dye™-800 (Rockland Immunochemicals, Gilbertsville, PA, USA) and AlexaFluor-680-conjugated antibodies (Invitrogen, Carlsbad, CA, USA) for Odyssey™ Infrared Imaging (Li-Cor, Lincoln, NE, USA). Dr Stephen P Ethier generously provided SUM cell RIPA lysates from the Karmanos Institute.

### siRNA transfection

Three siRNA oligonucleotides directed against each of XIAP and Survivin (Ambion) and a scrambled negative control siRNA (mock) were tested for effectiveness of protein knockdown. Transfection was performed with Lipofectamine 2000 (Invitrogen), according to the manufacturer's instructions. Transfections with a Cy3-conjugated scrambled negative control siRNA showed ~80% transfection efficiency in all cell lines used.

### Drug treatment

Cells were treated with TRAIL (10 ng/ml; Alexis Biochemicals, Grunberg, Germany), Trastuzumab (Herceptin^®^, 100 μg/ml), Lapatinib (Tykerb, GW572016, 100 nM), Gefitinib (Iressa, 10 μM) or Smac mimetic (10 nM) (generously provided by X Wang, University of Texas Southwestern Medical Centre at Dallas, TX, USA) for 48 hours when examining effects on apoptosis and proliferation. When examining the effects of the drugs on Erk signalling, cells were serum starved for a minimum of 4 hours prior to addition of the drugs for an additional 24 hours. Cells were then stimulated with epidermal growth factor (100 ng/ml) for 15 minutes at 37°C and lysed.

### Apoptosis and proliferation measurements

Cells were spun onto polysine^®^-coated slides, fixed in 4% formaldehyde and permeabilised in 0.2% Triton X-100. Nonspecific binding sites were blocked in 10% goat serum in PBS prior to staining for cleaved caspase 3 and the proliferation marker Ki67. Cells were co-stained with 4',6 diamidino-2-phenylindole (DAPI) and were viewed on an Axioplan2 microscope (Carl Zeiss MicroImaging Inc., Jena, Germany). Apoptosis was scored by examining nuclear morphology or caspase 3 staining. Counts were performed blind to prevent any bias, and at least 500 cells over two or more fields of view were counted for each sample. The cell turnover index (CTI) was calculated by dividing the percentage of proliferation by the percentage of apoptosis [33].

### Statistical analysis

A two-way analysis of variance was used. *P *< 0.05 indicated significance. Results are presented as the mean ± standard error of the mean from at least three experiments, unless stated otherwise.

## Results

### Inhibitor of apoptosis expression in common breast cancer cell lines

We examined the expression profile of the IAP family in a panel of commonly used breast cancer cell lines, together with the nonmalignant MCF10a cell line.

**Figure 1:**
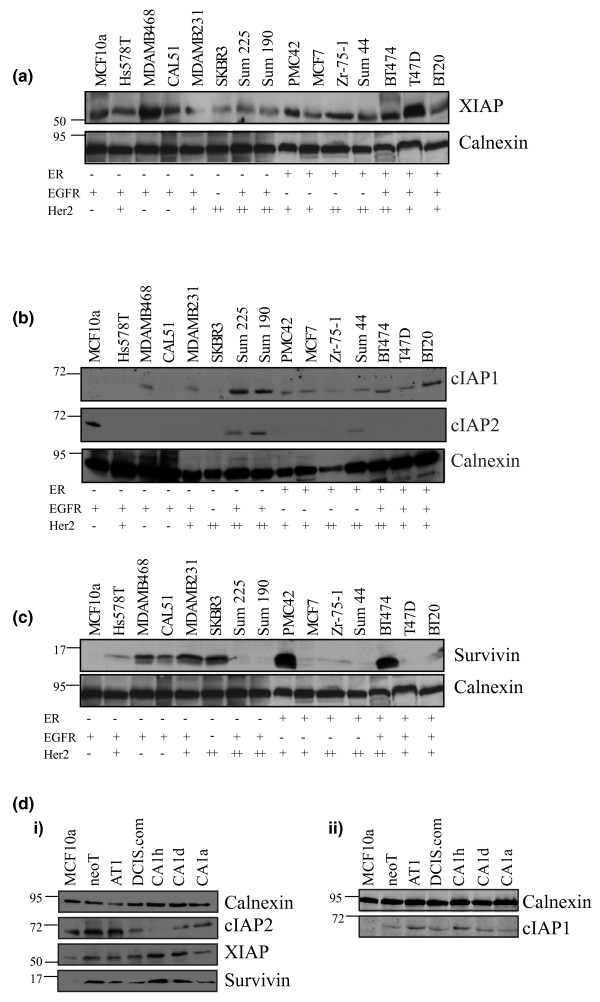
Inhibitor of apoptosis levels in breast cancer. Inhibitor of apoptosis (IAP) expression in a panel of normal and breast-cancer-derived cell lines was examined by immunoblotting with antibodies for **(a) **XIAP, **(b) **cIAP1 and cIAP2 (blotted sequentially) and **(c) **Survivin. Survivin and XIAP were detected on the same blot, which was stripped and reprobed for calnexin. **(d) **IAP expression was also determined in the MCF10 progression panel: **(i) **cIAP2, XIAP and Survivin and **(ii) **cIAP1. Representative blots from four experiments for the cell lines are shown. The receptor status of each cell line, determined by western blotting, is shown; there is no correlation with IAP expression. ER, oestrogen receptor; EGFR, epidermal growth factor receptor.

XIAP was ubiquitously expressed, but its levels varied across the panel (Figure 1a). Compared with MCF10a cells, XIAP was higher in only MDAMB468 and T47D cells. In contrast, cIAP1 was not detected in MCF10a cells but was markedly upregulated in the Sum 225, Sum 190 and BT20 cells compared with the MCF10a nonmalignant cell line (Figure 1b). On the other hand, cIAP2 was frequently expressed at lower levels in the cancer cell lines than in MCF10a cells. cIAP2 could be detected, however, in the Sum 225, Sum 190, and Sum 44 cell lines. Longer exposure times also showed cIAP2 was found in the MDAMB468, Zr-75-1 and T47D cell lines (Additional data file 1).

In contrast to the variable expression of XIAP, Survivin was elevated in about one-half of the cell lines examined in the present study while none was detected in the control MCF10a cell line. Marked increases in Survivin were seen in MDAMB468 cells, CAL51 cells, MDAMB231 cells, SKBR3 cells, PMC42 cells and BT474 cells compared with expression in MCF10a cells (Figure 1c).

Since the cell lines used above are from disparate sources, we also examined IAP levels in a separate panel of isogenic breast cell lines that shows increasing severity of tumour phenotype. This is the MCF10 progression panel, which includes the normal immortalised MCF10a cells, Ha-Ras transformed MCF10neoT cells, premalignant MCF10AT1 cells, a cloned xenograft lesion of MCF10AT1 cells (MCF10DCIS.com), and malignant variants that form invasive tumours with varying degrees of differentiation in xenografts (MCF10CA1, h, d, and a) [34,35].

In comparison with the parental MCF10a cells, Survivin, XIAP and cIAP1 were upregulated in all the transformed cells (Figure 1di, dii). cIAP2 levels appeared to decrease as the cell lines became more malignant. Interestingly, elevated Survivin, XIAP and cIAP1 levels were detected in cells corresponding to the early stages of breast cancer progression, in atypias (MCF10neoT and MCF10AT1 cells) and in MCF10DCIS-like cells.

### Knockdown of XIAP sensitises cells to TRAIL

**Figure 2:**
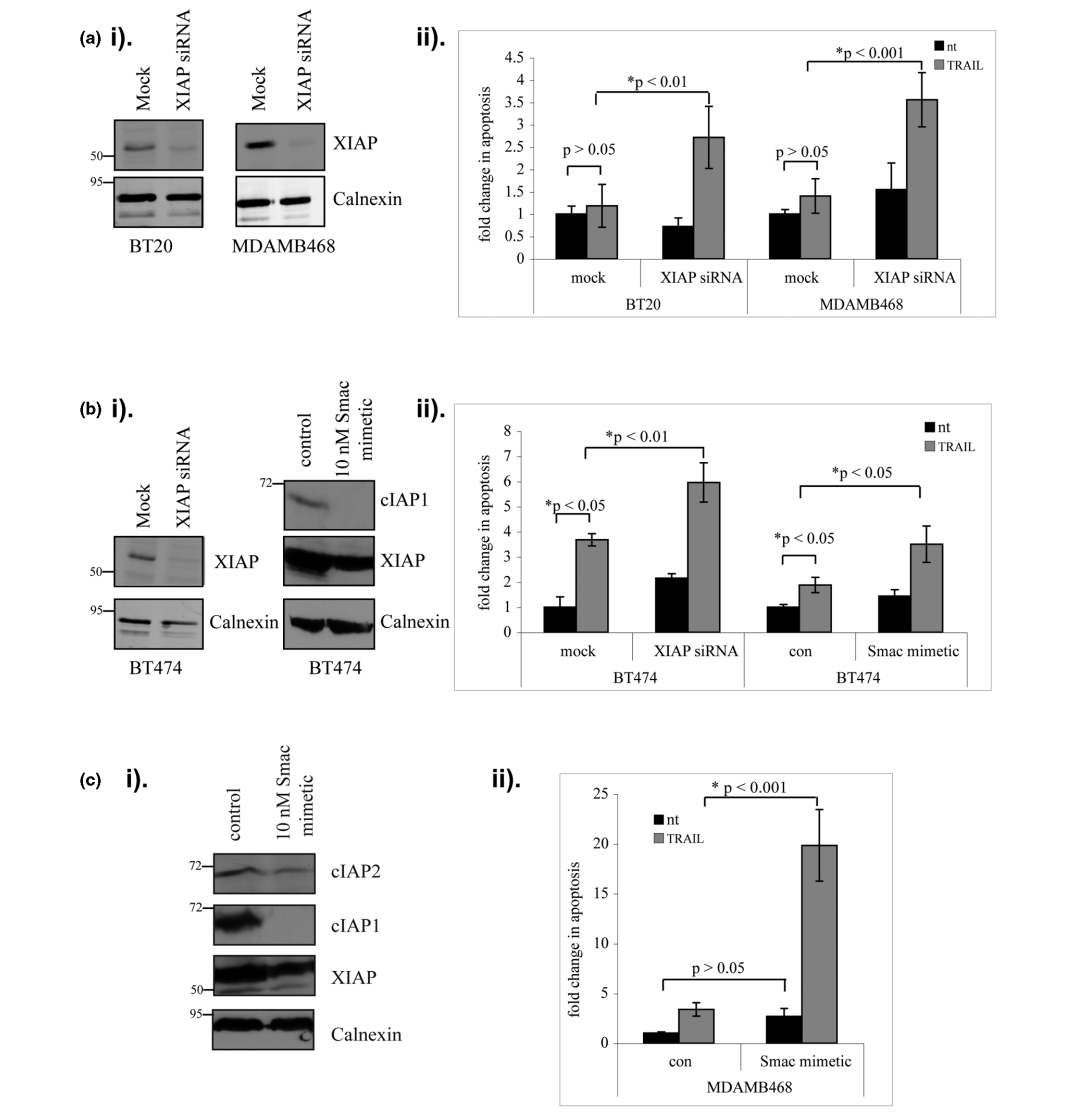
Inhibitor of apoptosis suppression sensitises cells to TRAIL-induced apoptosis. **(a) **BT20 and MDAMB468 cells were transfected with siRNA targeting XIAP, and 24 hours later cells were treated with TNF-related apoptosis-inducing ligand (TRAIL) (10 ng/ml) for 48 hours. Cells were then either **(i) **analysed by western blotting to confirm knockdown or **(ii) **cytospun and scored for apoptosis by staining for nuclear morphology. Data presented as fold changes in apoptosis (mean ± standard error of the mean (SEM)) from at least three independent experiments. **(b) **BT474 cells were either transfected with siRNA targeting XIAP and drug treated as above or treated with the Smac mimetic (10 nM) for 2 hours prior to TRAIL (10 ng/ml) addition for 48 hours: **(i) **knockdown was confirmed by western blotting (cIAP1 only, as cIAP2 was not detected in BT474 cells), and **(ii) **apoptosis was scored by examining nuclear morphology. Data presented as fold changes in apoptosis (mean ± SEM) from at least three independent experiments. **(c) **Effect of the Smac mimetic on MDAMB468 cells, examined as for BT474 cells in (b). Data presented as mean ± SEM from at least three experiments. nt, not treated; con, no IAP inhibitor.

Since XIAP is the most potent caspase inhibitor in the IAP family, we determined whether it contributed to the apoptotic resistance of breast cancer cells using RNAi. Each of three separate siRNAs targeting XIAP diminished its protein expression, and the sequence chosen for further studies reduced XIAP levels by greater than 80% in all cell lines used (Figure 2ai, bi). XIAP knockdown did not result in any significant changes in levels of the closely related cIAPs or Survivin (data not shown). The data obtained in the subsequent experiments were confirmed using a second siRNA sequence to rule out off-target effects (Additional data file 2).

To show that XIAP antagonism augments drug-induced apoptosis in breast cancer cells, we initially examined its effects in conjunction with TRAIL [26,36]. Currently in clinical trials for colon cancer, TRAIL initiates apoptosis through death receptor (DR4 or DR5)-induced activation of caspase 8. This pathway culminates in the activation of caspases 3 and 7, which are in turn inhibited by XIAP. The combination of TRAIL and XIAP antagonism was investigated in MDAMB468 cells, which express higher levels of XIAP than MCF10a cells, as well as BT20 and BT474 cells, which have similar or lower levels of XIAP (Figure 1a).

BT20 and MDAMB468 cells were insensitive to TRAIL (10 ng/ml), as shown by the lack of TRAIL-induced apoptosis in mock-transfected cells (Figure 2aii, compare black and grey columns in mock samples; the apoptosis data presented were determined by examining nuclear morphology, but in all cases similar results were obtained when cells were stained for cleaved caspase 3). Knockdown of XIAP significantly increased TRAIL-induced apoptosis in BT20 and MDAMB468 cells by 2.3-fold and 2.5-fold, respectively (Figure 2aii, compare grey columns between mock and siRNA transfected samples for each cell line).

In BT474 cells, which were initially sensitive to TRAIL-induced apoptosis (Figure 2bii, compare black and grey columns in mock transfected samples), knockdown of XIAP also resulted in a significant increase in TRAIL-induced apoptosis (Figure 2bii, compare grey columns between mock and XIAP siRNA samples). As well as targeting XIAP alone, we used a Smac mimetic (compound 3) that targets multiple IAPs by preventing the XIAP-mediated suppression of caspase activity and depleting the cells of cIAP1 and cIAP2 [37,38]. Smac mimetic (10 nM, 48 hours) resulted in complete depletion of cIAP1, and in a slight decrease in XIAP levels (Figure 2bi), but had no significant apoptotic effect on any of the cell lines in the absence of an apoptosis inducer (Figure 2bii, compare black columns (nt) between no inhibitor samples (con) and Smac mimetic samples). However, the Smac mimetic potentiated TRAIL-induced apoptosis compared with TRAIL treatment alone (1.7-fold), confirming the cooperative effect of IAP antagonists with TRAIL (Figure 2bii, compare grey columns for no inhibitor samples (con) versus Smac mimetic-treated samples).

In MDAMB468 cells, Smac induced depletion of cIAP1 and also decreased levels of cIAP2 (Figure 2ci). The effect of the Smac mimetic on MDAMB468 cells was more pronounced, causing a 5.8-fold increase in TRAIL-induced apoptosis (Figure 2c, compare grey columns in no inhibitor (con) samples versus Smac mimetic-treated samples). Since a much greater effect was seen with the Smac mimetic than with siRNA XIAP (compare Figures 2c and 2aii), this suggests that the cIAPs may play a key role in the resistance to TRAIL seen in MDAMB468 cells. Interestingly, the apoptotic effect of the Smac mimetic and TRAIL was specific to cancer cell lines as neither Smac nor TRAIL, alone or in combination, had any effect on the nonmalignant MCF10a cell line (Additional data file 3).

Regardless of the levels of XIAP in the cell lines, therefore, inhibiting XIAP either overcame the resistance to TRAIL (BT20 and MDAMB468 cells) or showed a synergistic increase in the apoptotic response to TRAIL (BT474 cells). Moreover, in MDAMB468 cells, targeting multiple IAPs in combination with TRAIL treatment produced a profound increase in apoptosis compared with targeting XIAP alone.

### Targeting XIAP sensitises cells to ErbB antagonists

A current strategy to treat cancer is via targeted drugs that antagonise oncogenically activated growth factor receptors. Many breast cancers overexpress Her2 (20% of all cases) and the EGFR. Targeting these receptors, to which the cancer cells have become addicted for survival, should block proliferation and either induce or sensitise the cells to apoptosis via the intrinsic pathway, thus providing the rational for using EGFR and Her2 in breast cancer therapy. Trastuzumab is a humanised monoclonal antibody, which inhibits Her2 and decreases proliferation in Her2-overexpressing cells such as BT474 [39]. Lapatinib is a dual kinase inhibitor of EGFR and Her2, while Gefitinib is a selective EGFR kinase inhibitor (but at higher concentrations, Gefitinib inhibits both the EGFR and Her2) [40,41].

**Figure 3:**
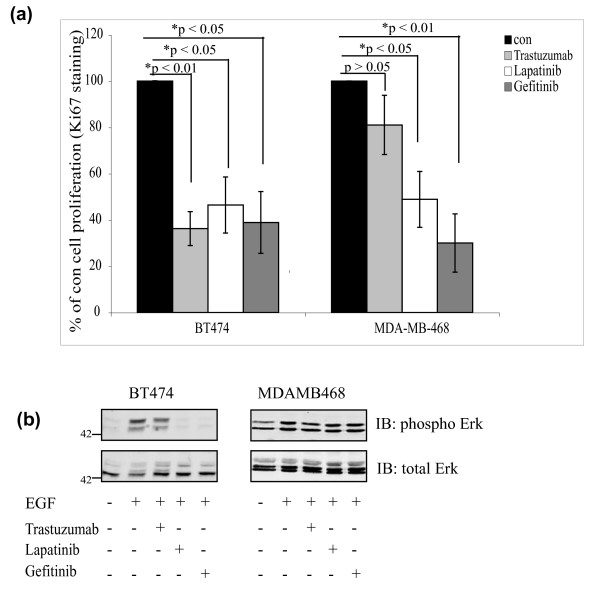
ErbB antagonists inhibit growth factor-induced proliferation and signalling. **(a) **The anti-proliferative effect of the ErbB antagonists was confirmed in BT474 and MDAMB468 cells, using Ki67 staining as a proliferation marker (48 hours post drug treatment). Data presented as mean ± standard error of the mean from at least three experiments. **(b) **Cells were serum-starved for 4 hours prior to the addition of vehicle only (con), Trastuzumab (100 μg/ml), Lapatinib (100 nM) or Gefitinib (10 μM) for 24 hours. Cells were stimulated with epidermal growth factor (EGF) (100 ng/ml) for 15 minutes at 37°C. Activation of Erk was detected by immunoblotting (IB).

To determine whether targeting IAPs in combination with these ErbB antagonists increased apoptosis, we used the Her2-overexpressing BT474 cells, which are predicted to be sensitive to inhibition by all three antagonists, and used the EGFR-overexpressing MDAMB468 cells, which should be sensitive to Gefitinib and Lapatinib but not Trastuzumab. Consistent with these ErbB receptor profiles, all three drugs significantly blocked proliferation in BT474 cells, and only Lapatinib and Gefitinib inhibited proliferation in the MDAMB468 cells (Figure 3a). We found that Lapatinib and Gefitinib inhibited Erk activation downstream of the EGFR, in BT474 cells, although Trastuzumab (as previously reported) was unable to do so (Figure 3b) [42]. MDAMB468 cells showed resistance to both Gefitinib and Lapatinib at the level of Erk phosphorylation [41,43].

**Figure 4:**
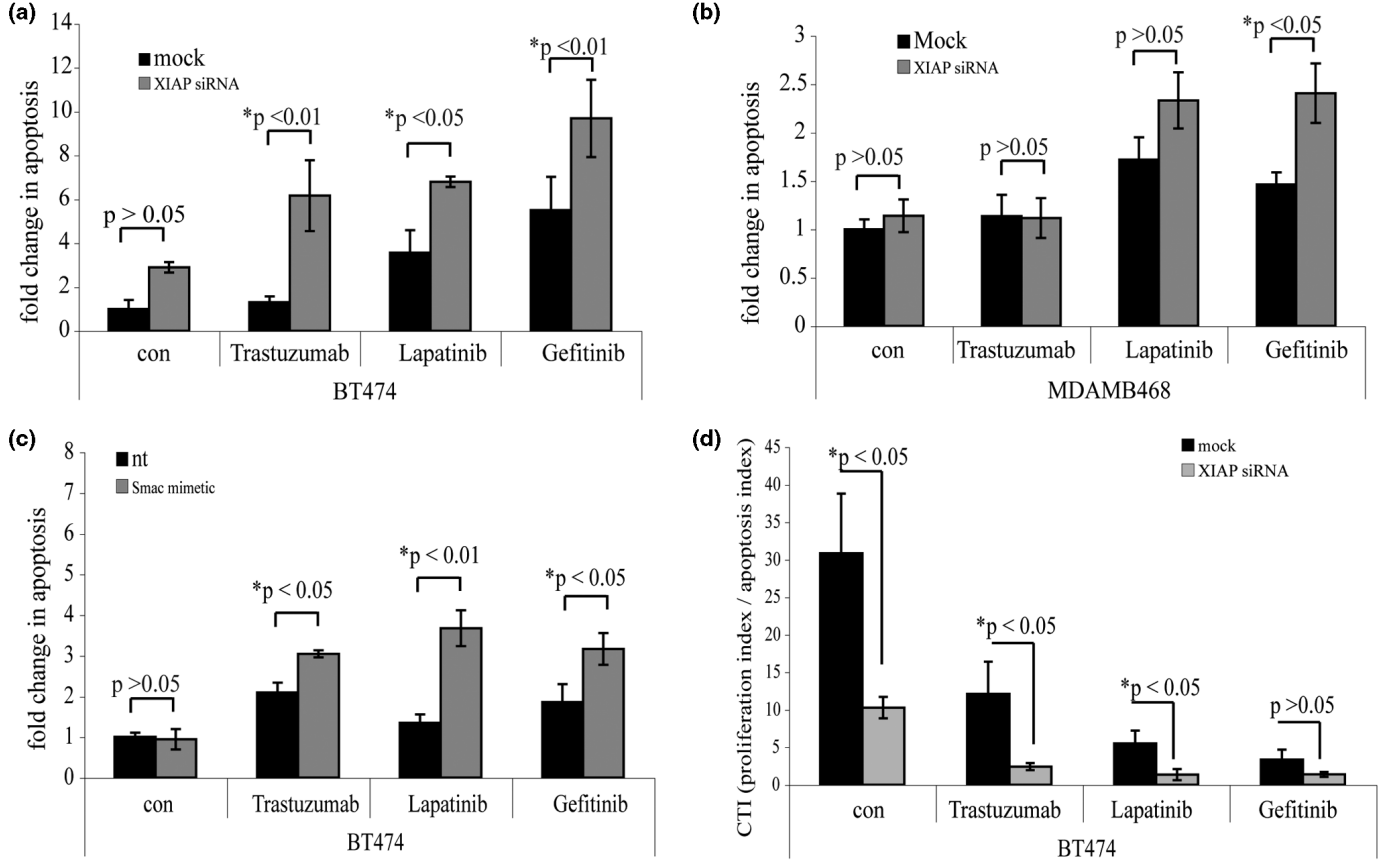
Targeting inhibitors of apoptosis increases sensitivity to ErbB antagonists. **(a) **BT474 cells and **(b) **MDAMB468 cells were transfected with siRNA targeting XIAP, and 24 hours later were treated with Trastuzumab (100 μg/ml), Lapatinib (100 nM), or Gefitinib (10 μM) for 48 hours. Apoptosis was scored, by examining nuclear morphology. Data presented as fold changes in apoptosis (mean ± standard error of the mean (SEM)) from at least three experiments. **(c) **BT474 cells were pretreated with the Smac mimetic for 2 hours before addition of Trastuzumab (100 μg/ml), Lapatinib (100 nM), or Gefitinib (10 μM). Cells were examined for nuclear morphology 48 hours later. Data presented as fold changes (mean ± SEM) from at least three experiments. **(d) **Cell turnover indexes (CTIs) for ErbB antagonist-treated BT474 cells in the presence or absence of XIAP depletion. Data presented as mean ± SEM from at least three experiments. *P values indicating significance. nt, not treated; con, no ErbB antagonist.

There is evidence that, in addition to blocking proliferation, these ErbB antagonists sensitise cells to apoptosis [42,44-46]. For example, Trastuzumab promotes apoptosis of breast cancer cells *in vivo*, and we have previously shown that Gefitinib induces apoptosis of normal breast epithelia through the intrinsic apoptosis pathway [46-49]. In BT474 and MDAMB468 cells, however, neither Trastuzumab, Lapatinib nor Gefitinib significantly elevated apoptosis above background (Figure 4a, b, c, compare black columns). We therefore reasoned that downregulating IAPs might sensitise the cells to undergo apoptosis in response to ErbB antagonists.

In control experiments XIAP knockdown had no significant effect on basal rates of apoptosis in any of the cell lines (Figure 4a, b, compare grey with black columns in no inhibitor samples). In addition, depleting XIAP by siRNA had no effect on the proliferation of any of the lines either in the presence or absence of the ErbB therapies (data not shown). XIAP depletion did, however, sensitise the cells to ErbB antagonist-induced apoptosis. This was most apparent in the ErbB2-overexpressing BT474 cells, where XIAP knockdown induced significant increases in response to Trastuzumab, Lapatinib and Gefitinib (4.7-fold, 1.9-fold, and 1.8-fold, respectively) (Figure 4a, compare grey with black columns in each drug-treated sample). In the EGFR-only overexpressing MDAMB468 cells, XIAP depletion significantly increased apoptosis in response to Gefitinib (1.7-fold) (Figure 4b, compare grey with black columns in each drug-treated sample).

We also examined whether the Smac mimetic enhanced the apoptotic effect of the ErbB antagonists. In the BT474 cell line, similar data to that seen with XIAP depletion were obtained with the Smac mimetic, which caused statistically significant increases in apoptosis induced by Trastuzumab, Lapatinib and Gefitinib (1.5-fold, 2.6-fold, and 1.5-fold, respectively) (Figure 4c, compare grey with black columns in each drug-treated sample).

The apoptotic response of some breast cancer cell lines to targeted therapies is therefore enhanced either by siRNA-mediated depletion of XIAP or by targeting multiple IAPs with a Smac mimetic. Although the overall amounts of apoptosis are modest following combined IAP suppression and ErbB antagonism, they are still relevant. Indeed, even small increases in apoptosis can have large effects on the CTI. The CTI measures the turnover of cells in a tumour, accounting for alterations in both proliferation and apoptosis. In BT474 cells, Trastuzumab, Lapatinib or Gefitinib induced significant decreases in the CTI of 2.5-fold, 5.5-fold, and 9.2-fold, respectively (Figure 4d, compare black columns). Importantly, the CTI was decreased by a further fivefold, fourfold, and 2.4-fold, respectively, upon depletion of XIAP (Figure 4d, compare grey with black columns), resulting in combined decreases over untreated controls of 12.7-fold, 23-fold, and 22.4-fold, respectively. As with XIAP knockdown, treatment with the Smac mimetic also reduced the CTI compared with drug treatment alone (data not shown).

Together, the increase in drug-induced apoptosis and the corresponding decrease in the CTI caused by IAP inhibition in BT474 and MDAMB468 cells demonstrate that IAPs can mediate resistance to ErbB antagonist-induced apoptosis in breast cancer cell lines. Targeting IAPs may therefore be important for use in combination therapies in the clinic.

### Survivin, and XIAP are upregulated in breast cancer biopsies

To examine IAP levels in breast cancer, we examined their expression at the protein level in biopsies of 11 or 12 breast tumours. Tumour samples were chosen to represent the major breast cancer subtypes: oestrogen receptor-positive and progesterone receptor-positive, oestrogen receptor-positive and progesterone receptor-negative, oestrogen receptor/progesterone receptor/ErbB2-positive, and oestrogen receptor-negative and progesterone receptor-negative (Additional data file 4). The IAP levels were compared with those in normal tissue samples obtained from reduction mammoplasties.

**Figure 5:**
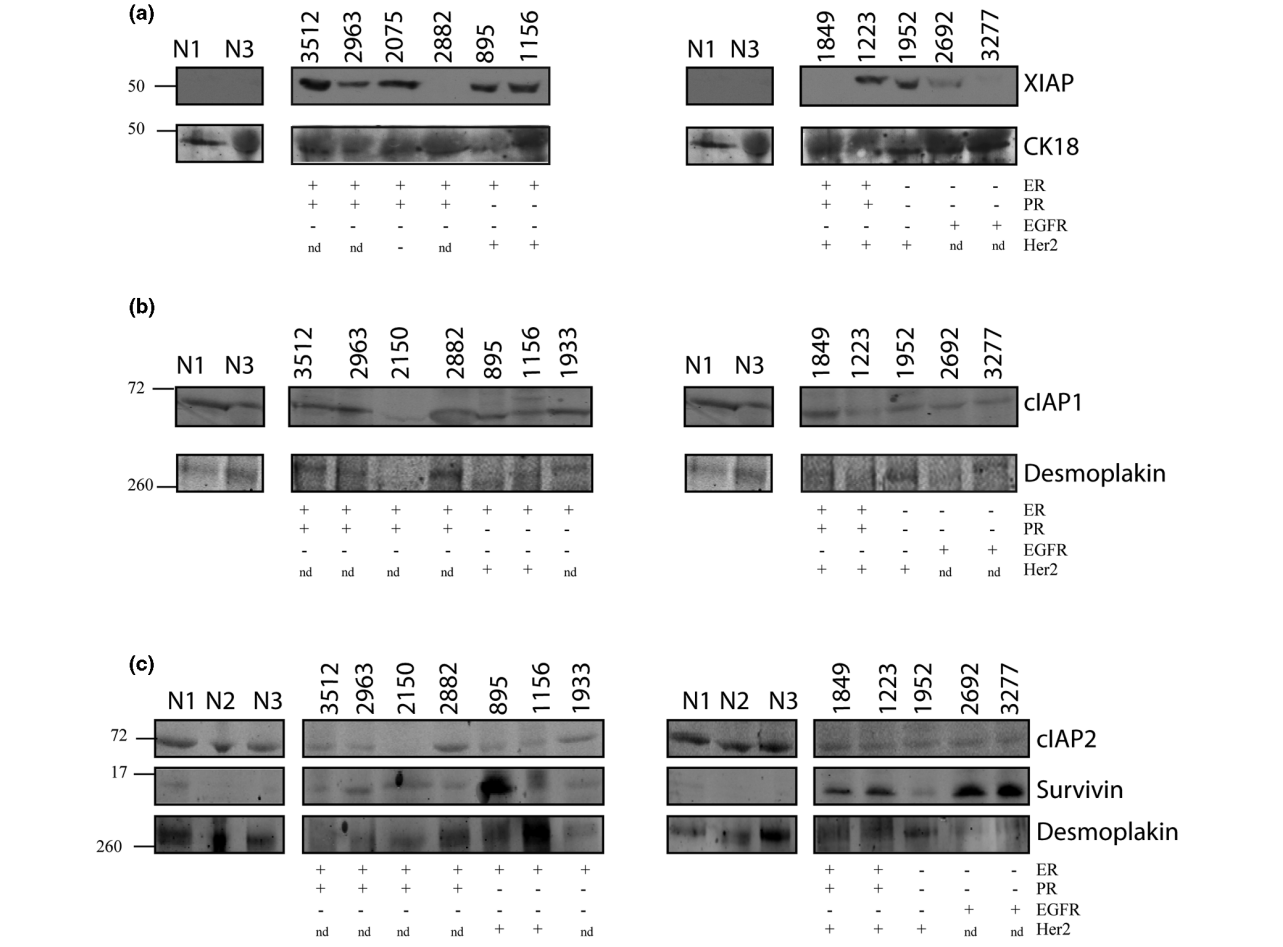
Inhibitor of apoptosis levels in patient samples. Inhibitor of apoptosis (IAP) levels in breast cancer biopsy samples and in samples of normal breast (N1 to N3) from reduction mammoplasties were examined by immunoblotting with the relevant antibodies. **(a) **XIAP was detected using enhanced chemiluminescence, blots were then stripped and reprobed for cytokeratin 18 (CK18). **(b) **cIAP1 and **(c) **cIAP2 and Survivin were detected using the Li-Cor Odyssey™ system. Blots were simultaneously probed for Desmoplakin. Prognostic indicators were determined in the clinic. ER, oestrogen receptor; EGFR, epidermal growth factor receptor; PR, progesterone receptor; +, positive; -, negative; nd, not determined.

XIAP was not detected in the normal tissue samples examined, and by comparison was elevated in eight out of 11 tumours (numbers 3512, 2963, 2075, 895, 1156, 1223, 1952 and 2692) (Figure 5a). cIAP1, however, was present in both normal and tumour tissue samples at relatively equal levels (Figure 5b). cIAP2 appeared to be at higher levels in the normal breast tissue samples compared with in the breast tumour samples (Figure 5c), mirroring what was seen in the cell line data. Survivin was upregulated in five out of the 12 tumour samples (numbers 895, 1849, 1223, 2692 and 3277), whereas it was absent in the blots of normal tissue samples (Figure 5c).

These data show that despite the variability in XIAP levels in the cell line panel, some patient samples show a marked upregulation of IAPs – and in fact multiple IAPs are upregulated in some cases (numbers 895, 1156). Also, since the pattern of IAP upregulation varies between tumour samples, any IAP-based therapy is going to need to be targeted at the correct IAP – and in some cases multiple IAPs – in a patient-specific manner.

## Discussion

The most important finding of the present study is that IAP antagonists in combination with clinically relevant ErbB family therapeutics promote apoptosis and dramatically reduce the CTI of breast cancers. We would therefore argue that, together with appropriate biomarkers, treating certain patients with IAP and ErbB antagonists together could be of clinical value.

We also present a note of caution, however, because some cell lines such as BT20 cells were responsive to treatment with IAP antagonists combined with TRAIL, but not the ErbB antagonists (data not shown).

### Inhibitor of apoptosis expression in breast cancer cell lines and biopsy samples

The upregulation of Survivin both in the breast cancer biopsy and in cell line panels is consistent with studies that have shown 71% of breast cancers were positive for Survivin, while the surrounding tissue was negative [50]. Survivin overexpression in tumours also seemed to correlate with Her2 overexpression, consistent with previous studies [51]. Of crucial importance in Survivin overexpression is its localisation, because nuclear Survivin indicates a good prognosis for recurrence-free survival in breast cancer, while cytoplasmic Survivin has a poorer outcome [13,50,52,53]. We have previously shown that MDAMB468 cells express both nuclear and cytoplasmic Survivin, thereby reflecting what occurs *in vivo*, and that cytoplasmic Survivin has an anti-apoptotic role [54]. Despite a number of anti-Survivin therapies currently showing promise in clinical trails, however, targeting of Survivin with siRNA in the BT474 cells did not elevate their apoptotic sensitivity to TRAIL or to the ErbB antagonists Lapatinib or Gefitinib (Additional data file 5).

XIAP is the most potent caspase inhibitor of all of the IAPs, and blocks both the intrinsic and extrinsic apoptosis pathways by binding to caspases 3, 7 and 9. Currently there are surprisingly few studies on XIAP in breast cancer, and these few have focused on a limited number of cell lines; to our knowledge, only one other study has examined its *in vivo *expression in comparison with normal breast, where XIAP positivity correlated with tumour grade [2,25]. As with previous studies in the breast cancer cell lines and in the National Cancer Institute (NCI) panel of tumour cell lines, we found that XIAP expression was variable [16,26]. This variability possibly reflects the multiple mechanisms by which XIAP can be regulated, all of which or some of which may be altered in cancer. Despite this variability in the cell line panel, XIAP was more markedly upregulated in a subset of the breast tumour samples compared with normal tissue.

In the case of cIAP1, there was a marked upregulation in the cancer cell lines compared with MCF10a cells. No such upregulation was seen, however, in the tumour panel. The present study is the first that has examined cIAP1 protein in breast tumour biopsies and compared its expression between normal breast epithelia and breast cancer samples or cell lines. Interestingly, we found that cIAP2 was lower in the cancer cell lines versus normal cell lines (confirmed with a second anti-cIAP2 antibody) and in the tumour tissue versus normal tissue samples. Future studies involving greater patient numbers with matched cancer and normal tissue samples are required to confirm the variation in cIAP1 and the possible downregulation of cIAP2 in invasive carcinomas.

Overall, these data demonstrate that multiple IAPs are expressed in breast cancer and therefore are potentially responsible for apoptotic resistance. IAPs, in particular XIAP and cIAPs, are therefore potential targets for lowering the apoptotic threshold of breast cancers, making them more sensitive to therapeutic drugs. We confirmed this hypothesis by examining TRAIL-induced apoptosis in the presence or absence of an XIAP siRNA or a Smac mimetic. Importantly, this had a major effect in one cell line – the MDAMB468 cells. The Smac mimetic has also been shown to substantially increase TRAIL-induced apoptosis in the MDAMB231 cell line [26]. Interestingly both the MDAMB468 and MDAMB231 cells are triple-negative (oestrogen receptor/progesterone receptor/ErbB2-negative) cell lines. In BT474 cells and BT20 cells, which did not appear to have elevated levels of XIAP, inhibition of XIAP also increased TRAIL-induced apoptosis, suggesting that XIAP does not need to be overexpressed for the Smac mimetic to be of use. Of potential clinical importance, neither TRAIL nor the Smac mimetic had any effect in the MCF10a cell line.

### Inhibitors of apoptosis and ErbB antagonists

Only a few studies have looked at combining XIAP inhibitors with targeted therapies to growth factor receptors. In human glioblastoma multiforme, combining the platelet-derived growth factor receptor antagonist Imatinib with a Smac mimetic significantly increased cell death compared with Imatinib treatment alone [55]. In breast cancer, Smac mimetics have been shown to increase the apoptotic effect of Tamoxifen in oestrogen receptor-overexpressing cell lines [30]. In inflammatory breast cancer, Trastuzumab treatment was shown to induce an upregulation of XIAP expression. When XIAP was targeted in these inflammatory breast cancer cell lines, a greater decrease in cell viability was observed in combination with Trastuzumab than with Trastuzumab treatment alone [31].

We have studied the combination of IAP targeting and growth factor receptor inhibition in breast cancer cell lines, where overexpression of the EGFR or Her2 is common. In these cell lines, treatment with the growth factor receptor antagonists did not induce changes in IAP expression levels (data not shown). We found that IAP inhibition alone did not affect the basal rates of apoptosis. In combination with a targeted therapy, however, IAP inhibition resulted in increased rates of apoptosis and substantially reduced the CTI. This indicates that IAP inhibition alone has no detrimental effect on cells, but would enhance apoptosis in cancer cells targeted by the breast-cancer-specific therapeutics. We therefore suggest that inhibiting IAPs may be a valuable adjunct to other therapies in a clinical setting.

Further research still needs to be done in order to determine what else, other than IAPs, might be contributing to the apoptotic resistance of breast cancer cells. Such factors possibly include members of the Bcl-2 family, which are upregulated in some breast cancers.

## Conclusions

The combination of IAP antagonists with drugs that target ErbB receptors promotes apoptosis and dramatically reduces the cell turnover index of some breast cancer cell lines. We suggest that treating certain patients with IAP and ErbB antagonists together could be of clinical benefit.

## Abbreviations

CTI: cell turnover index; DMEM: Dulbecco's modified Eagles' medium; DAPI: 4',6 diamidino-2-phenylindole; EGFR: epidermal growth factor receptor; FCS: foetal calf serum; IAP: inhibitor of apoptosis; PBS: phosphate-buffered saline; siRNA: small interfering RNA; Smac: second mitochondrial activator of caspases; TNF: tumour necrosis factor; TRAIL: TNF-related apoptosis-inducing ligand.

## Competing interests

The authors declare that they have no competing interests.

## Authors' contributions

FMF designed the experiments and performed the western blot analysis of the expression levels in the cell lines and tumours, the apoptosis and proliferation assays, interpreted the results, generated the figures and wrote the manuscript. TWO and JT-H lysed the tumour cell lines and validated the antibodies for IAP detection. RBC provided the human breast cancer samples and normal controls. KB provided the MCF10a progression cell line panel and many useful discussions on experimental designs. NJB and CHS conceived the project, and CHS oversaw the management of the project and contributed to writing the manuscript. All authors proofread, assisted in revision of and approved the final manuscript.

## Supplementary Material

Additional file 1 **Adobe file containing a figure that shows cIAP levels in breast cancer cell lines**. Relative migration positions of cIAP1 and cIAP2 were determined on the Li-Cor Odyssey™ system prior to samples being re-run and probed with enhanced chemiluminescence. A long exposure is shown, where MDAMB468, Sum225, Sum190 and Zr-75-1 cell lines show detectable cIAP2 levels, although these levels are still lower than that observed in the nonmalignant MCF10a cell line. Click here for file

Additional file 2 **Adobe file containing a figure that shows the effect of a second XIAP siRNA and the scrambled (mock) siRNA oligonucleotides on TRAIL (10 ng/ml) or Gefitinib (10 μM)-induced apoptosis in BT474 cells**. Data are presented as fold changes in apoptosis (mean ± standard error of the mean). nt: nontreated. Click here for file

Additional file 3 **Adobe file containing a figure that shows the effect of Smac mimetic on TRAIL-induced apoptosis in MCF10a cells. MCF10a cells were pretreated with the Smac mimetic for 2 hours prior to TRAIL (10 ng/ml) addition for 48 hours**. Data are presented as fold changes in apoptosis (mean ± standard error of the mean). nt: nontreated; con: no Smac mimetic. Click here for file

Additional file 4 **Adobe file containing a table that lists prognostic information on tumour samples**. Steroid receptor (oestrogen receptor percentage/progesterone receptor percentage), proliferative index (%Ki67), and growth factor receptor status (EGFR, C-erbB-2) as well as tumour type, grade and lymph node metastasis (number positive/number examined) were determined in the clinic. Click here for file

Additional file 5 **Adobe file containing a figure that shows the effect of Survivin knockdown on ErbB antagonist or TRAIL-induced apoptosis in BT474 cells**. Data presented as mean ± standard error of the mean. Click here for file
