# Supplementary figures and images for: Targeting inhibitor of apoptosis proteins in combination with ErbB antagonists in breast cancer
Source: Breast Cancer Res. 2009 Jun 29;11(3):R41. doi: 10.1186/bcr2328 (PMC2716510; doi:10.1186/bcr2328)

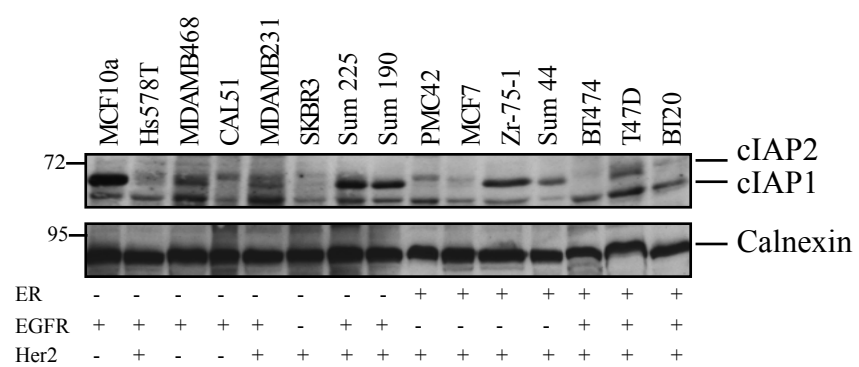

Foster et al. Figure S1: cIAP levels in breast cancer cell lines

Supplement: Additional file 1 — Adobe file containing a figure that shows cIAP levels in breast cancer cell lines. Relative migration positions of cIAP1 and cIAP2 were determined on the Li-Cor Odyssey™ system prior to samples being re-run and probed with enhanced chemiluminescence. A long exposure is shown, where MDAMB468, Sum225, Sum190 and Zr-75-1 cell lines show detectable cIAP2 levels, although these levels are still lower than that observed in the nonmalignant MCF10a cell line. [file bcr2328-S1.pdf]

**A.**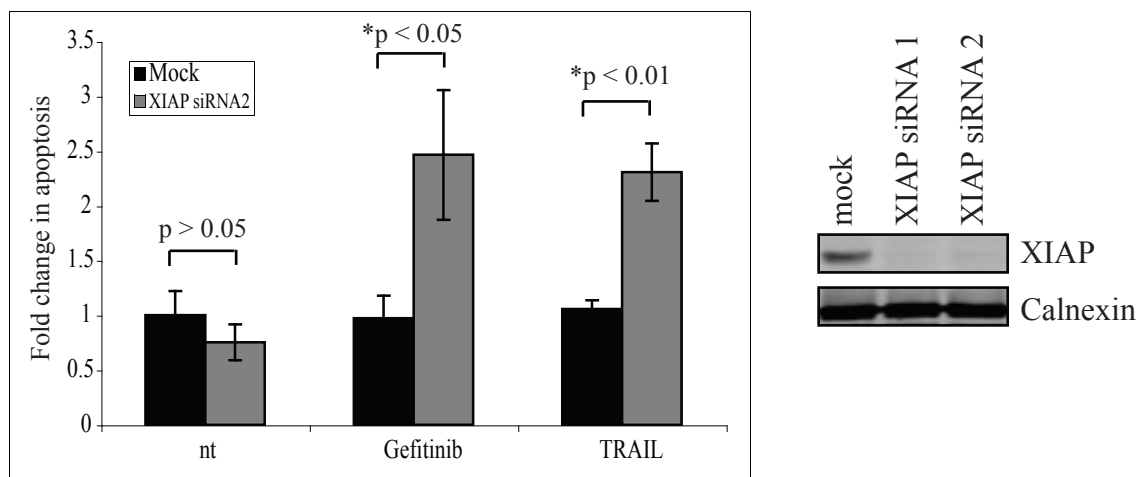

Supplement: Additional file 2 — Adobe file containing a figure that shows the effect of a second XIAP siRNA and the scrambled (mock) siRNA oligonucleotides on TRAIL (10 ng/ml) or Gefitinib (10 μM)-induced apoptosis in BT474 cells. Data are presented as fold changes in apoptosis (mean ± standard error of the mean). nt: nontreated. [file bcr2328-S2.pdf]

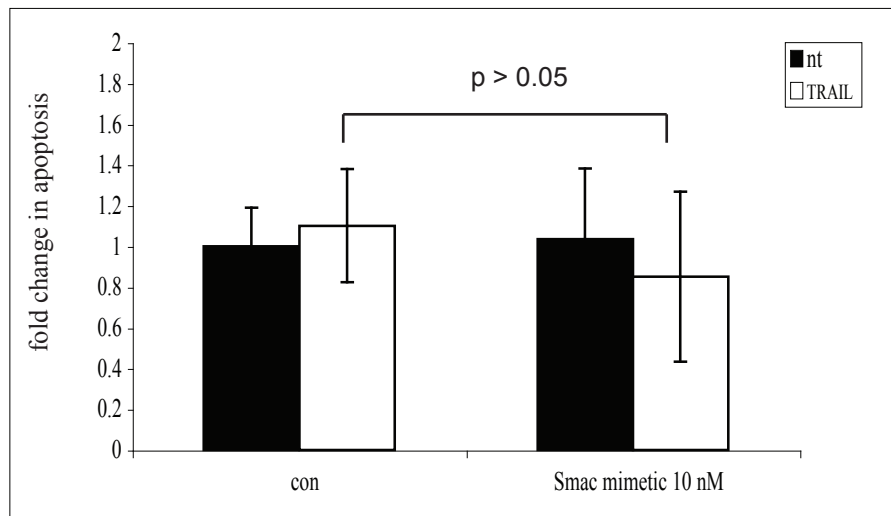

Foster et al. Figure S3: Effect of Smac mimetic on TRAIL-induced apoptosis in MCF10a cells.

Supplement: Additional file 3 — Adobe file containing a figure that shows the effect of Smac mimetic on TRAIL-induced apoptosis in MCF10a cells. MCF10a cells were pretreated with the Smac mimetic for 2 hours prior to TRAIL (10 ng/ml) addition for 48 hours. Data are presented as fold changes in apoptosis (mean ± standard error of the mean). nt: nontreated; con: no Smac mimetic. [file bcr2328-S3.pdf]

**A.**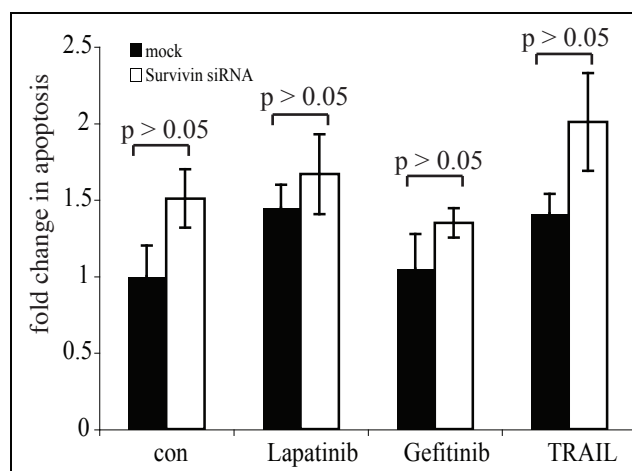**B.**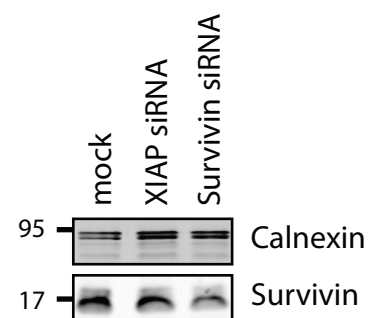

Foster et al. Figure S5: Effect of Survivin knock-down on ErbB antagonist-induced apoptosis

Supplement: Additional file 5 — Adobe file containing a figure that shows the effect of Survivin knockdown on ErbB antagonist or TRAIL-induced apoptosis in BT474 cells. Data presented as mean ± standard error of the mean. [file bcr2328-S5.pdf]
