# Supplementary material for: Targeting inhibitor of apoptosis proteins in combination with ErbB antagonists in breast cancer
Source: Breast Cancer Res. 2009 Jun 29;11(3):R41. doi: 10.1186/bcr2328 (PMC2716510; doi:10.1186/bcr2328)
Supplement: Additional file 4 — Adobe file containing a table that lists prognostic information on tumour samples. Steroid receptor (oestrogen receptor percentage/progesterone receptor percentage), proliferative index (%Ki67), and growth factor receptor status (EGFR, C-erbB-2) as well as tumour type, grade and lymph node metastasis (number positive/number examined) were determined in the clinic. [file bcr2328-S4.pdf]

LAB NO	ER, %	PR, %	Ki67/MIB1, %	EGFR, (Neg-4+)	C-erbB-2	TUMOUR TYPE	GRADE	LYMPH NODE METS
3512	99	93	8.9	NEG	nd	ductal	II	0/8
2963	96	96	9.9	NEG	nd	ductal	II	2/21
2075	51	10	15.6	NEG	NEG	ductal	nd	nd
2150	89	95	2.8	NEG	nd	lobular	II	0/18
2882	91	77	8.6	NEG	nd	Lob / ductal	I	0/18
895.2	40	2	26.8	NEG	1+	ductal	II	0/15
1156.4	70	0	20	NEG	1+	ductal	III	0/12
1933	65	0	0.9	NEG	nd	lobular	II	21/21
1849.1	34	48	33	NEG	3+	ductal	II	8/25
1223.2	90	15	37.6	NEG	3+	ductal	III	7/21
1952.1	0	0	5.2	NEG	3+	ductal	III	0/11
2692	0	0	27.8	3+	nd	ductal	III	4/18
3077.1	0	0	31.5	2+/3+	nd	ductal	III	nd

Foster et al. Figure S4: Prognostic Information on Tumour Samples
